# Comparative Genomic and Phylogenomic Analyses Clarify Relationships Within and Between *Bacillus cereus* and *Bacillus thuringiensis*: Proposal for the Recognition of Two *Bacillus thuringiensis* Genomovars

**DOI:** 10.3389/fmicb.2019.01978

**Published:** 2019-08-23

**Authors:** Inwoo Baek, Kihyun Lee, Michael Goodfellow, Jongsik Chun

**Affiliations:** ^1^School of Biological Sciences, Seoul National University, Seoul, South Korea; ^2^Institute of Molecular Biology and Genetics, Seoul National University, Seoul, South Korea; ^3^Department of Systems Biotechnology, Chung-Ang University, Anseong, South Korea; ^4^School of Natural and Environmental Sciences, Newcastle University, Newcastle upon Tyne, United Kingdom

**Keywords:** *Bacillus cereus*, *Bacillus thuringiensis*, Bt toxin, Cry toxin, phylogenomic analysis

## Abstract

The present study was designed to clarify the taxonomic status of two species classified as *Bacillus cereus sensu lato*, namely *B. cereus sensu stricto* and *Bacillus thuringiensis*. To this end, nearly 900 whole genome sequences of strains assigned to these taxa were the subject of comparative genomic and phylogenomic analyses. A phylogenomic tree based on core gene sequences showed that the type strains of *B. cereus* and *B. thuringiensis* formed a well-supported monophyletic clade that was clearly separated from corresponding clades composed of the remaining validly published species classified as *B. cereus sensu lato*. However, since average nucleotide identity and digital DNA–DNA hybridization similarities between the two types of *Bacillus* were slightly higher than the thresholds used to distinguish between closely related species we conclude that *B. cereus* and *B. thuringiensis* should continue to be recognized as validly published species. The *B. thuringiensis* strains were assigned to two genomically distinct groups, we propose that these taxa be recognized as genomovars, that is, as *B. thuringiensis* gv. *thuringiensis* and *B. thuringiensis* gv. *cytolyticus*. The extensive comparative genomic data clearly show that the distribution of pesticidal genes is irregular as strains identified as *B. thuringiensis* were assigned to several polyphyletic groups/subclades within the *B. cereus*–*B. thuringiensis* clade. Consequently, we recommend that genomic or equivalent molecular systematic features should be used to identify *B. thuringiensis* strains as the presence of pesticidal genes cannot be used as a diagnostic marker for this species. Comparative taxonomic studies are needed to find phenotypic properties that can be used to distinguish between the *B. thuringiensis* genomovars and between them and *B. cereus*.

## Introduction

*Bacillus cereus sensu lato*, also known as the *B. cereus* group, is a phylogenetically defined taxon within the genus *Bacillus* (Cohn, 1872) which encompasses an array of Gram-stain-positive, rod-shaped, facultatively anaerobic, endospore-forming bacteria that are common in natural habitats (Guinebretiere et al., 2013; Liu et al., 2017b; Patino-Navarrete and Sanchis, 2017). The group currently contains 21 validly published species (Liu et al., 2017a) which include *Bacillus anthracis* (Cohn, 1872), the causal agent of anthrax (Ezzell and Welkos, 1999; Moayeri et al., 2015); *B. cereus* (Frankland and Frankland, 1887), an opportunistic pathogen that causes food poisoning (Kotiranta et al., 2000; Bottone, 2010); *Bacillus thuringiensis* (Berliner, 1915), which produces insecticidal toxins widely used as biological control agents (Bravo et al., 2013; Raymond and Federici, 2017); and *Bacillus toyonensis* (Jimenez et al., 2013), which is used as a probiotic in animal nutrition. Members of these and related species assigned to the *B. cereus* group have been extensively studied given their economic and medical importance (Bottone, 2010; Bravo et al., 2013; Lacey et al., 2015; Hong et al., 2016; Kumari and Sarkar, 2016; Soni et al., 2016; Raymond and Federici, 2017).

*Bacillus cereus sensu stricto* is a common soil organism that is better known as a source of toxins associated with two forms of food poisoning, emesis and diarrhea. Emesis is caused by the toxin peptide cereulide that is encoded by *ces* genes located on a mega-virulence plasmid related to the *B. anthracis* toxin plasmid XO1 (Ehling-Schulz et al., 2004, 2005, 2015). Cereulide-producing *B. cereus* strains, in contrast to their diarrheal counterparts, form a single evolutionary lineage of closely related strains (Ehling-Schulz et al., 2006). Diarrheal food poisoning is caused by the single or combined action of heat-labile enterotoxins (Ehling-Schulz et al., 2004). In particular, three enterotoxins expressed by chromosomal genes (Fagerlund et al., 2010) are linked to this condition: the protein complexes hemolysin BL (Hbl), its non-hemolytic counterpart (Nhe), and the single protein cytotoxin K (CytK) (Stenfors Arnesen et al., 2008; Ceuppens et al., 2011). However, *cytK* and corresponding genes on the *hbl* operon are also evident in species of *B. cereus sensu lato* as a consequence of extensive lateral gene transfer events (Bohm et al., 2015); food poisoning toxicity can also be affected by transcription and unknown environmental factors (Jessberger et al., 2015). In turn, *B. thuringiensis* strains and associated parasporal crystal proteins are widely used as biological agents (Bt toxins) to control insect pests (Palma et al., 2014); the ability to synthesize crystal and cytotoxic enterotoxins are encoded by plasmid-borne *cry* and *cyt* genes, respectively (Schnepf et al., 1998; Palma et al., 2014). Many Bt toxins have been reported and classified based on amino acid sequences^1^ (Table 1; Crickmore et al., 1998; Berry and Crickmore, 2017).

**TABLE 1:** Pesticidal toxins discovered in the *B. cereus*–*B. thuringiensis* clade.

**Type**	**Structure**	**Type**	**Structure**	**Type**	**Structure**	**Type**	**Structure**
Cry1	3D	Cry21	3D	Cry41	3D	Cry61	3D
Cry2	3D	Cry22	Cry6 like	Cry42	3D	Cry62	3D
Cry3	3D	Cry23	Mtx	Cry43	3D	Cry63	3D
Cry4	3D	Cry24	3D	Cry44	3D	Cry64	Mtx
Cry5	3D	Cry25	3D	Cry45	Mtx	Cry65	3D
Cry6	Cry6 like	Cry26	3D	Cry46	Mtx	Cry66	3D
Cry7	3D	Cry27	3D	Cry47	3D	Cry67	3D
Cry8	3D	Cry28	3D	Cry48	3D	Cry68	3D
Cry9	3D	Cry29	3D	Cry49	Bin	Cry69	3D
Cry10	3D	Cry30	3D	Cry50	3D	Cry70	3D
Cry11	3D	Cry31	3D	Cry51	Mtx	Cry71	3D
Cry12	3D	Cry32	3D	Cry52	3D	Cry72	3D
Cry13	3D	Cry33	Mtx	Cry53	3D	Cry73	3D
Cry14	3D	Cry34	^∗^	Cry54	3D	Cry74	Mtx
Cry15	Mtx	Cry35	Bin	Cry55	^∗^	Cyt1	Cyt
Cry16	3D	Cry36	Bin	Cry56	3D	Cyt2	Cyt
Cry17	3D	Cry37	Cry6 like	Cry57	3D	Cyt3	Cyt
Cry18	3D	Cry38	Mtx	Cry58	3D	Vip1	Vip
Cry19	3D	Cry39	3D	Cry59	3D	Vip2	Vip
Cry20	3D	Cry40	3D	Cry60	Mtx	Vip3	^∗^
						Vip4	Vip

*Data are derived from Van Der Hoeven (2014) and Berry and Crickmore (2017). Features: 3D, 3-domain toxins; Mtx, insecticidal crystal protein that resembles the toxin of *Clostridium perfringens*; Bin, toxins that resemble the toxin of *Lysinibacillus sphaericus*; Cyt, insecticidal cytotoxic protein; Vip/Vip3, insecticidal vegetative protein; Cry6 like, Cry toxins with shorter sequences and distinct structures; and ^∗^, unique structural category.*

Phenotypic and genotypic approaches have been used to characterize species assigned to the *B. cereus* group. Phenotypic markers include biochemical (Logan and Berkeley, 1984), colonial (Flugge, 1886), and plasmid-encoded features (Rasko et al., 2005) and genotypic methods by various procedures, such as DNA–DNA hybridization (Hill et al., 2004), multilocus enzyme electrophoresis (Carlson et al., 1994; Vilas-Boas et al., 2002), pulsed-field gel electrophoresis (Zhong et al., 2007), and single (Ko et al., 2004; La Duc et al., 2004) and multilocus sequence analyses of conserved housekeeping genes (Helgason et al., 2004; Priest et al., 2004). Recently combinations of these two approaches have been used for classifying members of the *B. cereus* group (Jimenez et al., 2013; Liu et al., 2017a). Such studies have clarified relationships among species classified as *B. cereus sensu lato* (Liu et al., 2017b) even though problems remain, notably in distinguishing between *B. anthracis*, *B. cereus*, and *B. thuringiensis* (Patino-Navarrete and Sanchis, 2017). The historical distinction between these taxa based on plasmid expressed features is not reliable; the loss of *cry* genes from *B. thuringiensis* strains, for instance, makes them indistinguishable from *B. cereus sensu stricto* (Lin et al., 1998). Further, members of these taxa are very difficult to differentiate using genotypic criteria, as witnessed by the low genetic diversity seen from analyses of 16S rRNA (Daffonchio et al., 2006) and protein-coding gene sequences (Priest et al., 2004) while DNA–DNA hybridization values between representatives of these species have been reported to be above the 70% cut-off point used to circumscribe prokaryotic species (Liu et al., 2017a). Genotypic properties such as these have led to suggestions that these taxa be recognized as a single species (Helgason et al., 2000, 2004; Rasko et al., 2005) or as subspecies of *B. cereus sensu stricto* (Ash et al., 1991). However, in the meantime, *B. anthracis* has been distinguished from *B. cereus* and *B. thuringiensis* using several taxonomic procedures (Radnedge et al., 2003; Zhong et al., 2007; Patino-Navarrete and Sanchis, 2017).

Classifications based on whole-genome sequences and associated bioinformatic tools are not only clarifying relationships between closely related bacteria that proved difficult to resolve using conventional taxonomic procedures (Carro et al., 2018; Gupta et al., 2018; Nouioui et al., 2018; Sangal et al., 2018) but are also providing improved metrics for recognizing species boundaries (Chun and Rainey, 2014; Chun et al., 2018), such as those based on average nucleotide identity (ANI; Goris et al., 2007; Arahal, 2014) and digital DNA–DNA hybridization (dDDH; Auch et al., 2010; Meier-Kolthoff et al., 2013). Similarly, dDDH values of 79–80% have been set for the recognition of sub-species (Meier-Kolthoff et al., 2014; Nouioui et al., 2018). *B. cereus sensu lato* species fall below the ANI and dDDH thresholds for separating species (95–96 and 70%, respectively), apart from *B. cereus sensu stricto* and *B. thuringiensis* (96.8 and 71.2%) and *Bacillus mycoides* and *Bacillus weihenstephanensis* (97.6 and 78.2% (Liu et al., 2017a). The latter is now considered to be a later heterotypic synonym of *B. mycoides* (Liu et al., 2018), but the relationship between *B. cereus* and *B. thuringiensis* has still to be resolved. However, it is now clear that the presence or absence of plasmid-bearing genes cannot be used to separate these taxa (Liu et al., 2015), a result in agreement to those of earlier studies (Kolsto et al., 2009; Zwick et al., 2012).

The present study was designed to determine the taxonomic relationship between *B. cereus* and *B. thuringiensis* based on comparisons of high-quality whole-genome sequences of nearly 900 strains. By linking phylogenomic relationships and the distribution of genes encoding toxin and other taxonomic markers, we propose that the *bona fide* members of *B. cereus* and *B. thuringiensis* be classified into three genomically coherent groups, *B. cereus*, *B. thuringiensis* genomovar *thuringiensis*, and *B. thuringiensis* genomovar *cytolyticus*; emended descriptions are given of *B. cereus* and *B. thuringiensis*.

## Materials and Methods

### Genome Data Acquisition and Filtering Out Low-Quality Genomes

Genome sequences and corresponding annotated protein sequences of the 973 *B. cereus* and *B. thuringiensis* strains were downloaded from the EzBioCloud database (Yoon et al., 2017a; Supplementary Table S1). Genomes with either >500 contigs or an N50 size under 20,000 bp were excluded from the dataset as they were considered to be poorly assembled, as were genomes found by CheckM to be either incomplete (<95%) or had a contamination rate above 5% (Parks et al., 2015). The completeness of the genomes was also checked using bacterial core genes extracted by UBCG software (v.3.0^2^; Na et al., 2018); when >5% of the 92 core gene set was absent the genomes were excluded from further analysis. Outlier strains with long branches were removed by TreeShrink (Mai and Mirarab, 2018) from the FastTree (Price et al., 2010)-driven phylogenomic tree. The resultant 898 strains were the subject of further studies.

### Phylogenomic Tree Reconstruction of the *Bacillus cereus*–*Bacillus thuringiensis* Clade

Based on the core genes extracted using the UBCG software, a maximum-likelihood phylogenomic tree of the filtered *B. cereus* and *B. thuringiensis* strains was constructed by IQ-TREE version 1.6.7 (Nguyen et al., 2015) with 1000 bootstrap replications (Minh et al., 2013; Hoang et al., 2018). The model used for the phylogenetic estimations was automatically detected as the GTR + F + R10 model by using the ModelFinder algorithm implemented in IQ-TREE (Kalyaanamoorthy et al., 2017). UBCG software executed with the RAxML (v. 8.2.8) option was used to generate a more rigorous maximum-likelihood phylogenomic tree which included genomes of the type species belonging to the *B. cereus* group and related taxa (Stamatakis, 2014; Na et al., 2018). The iTOL v3 webserver^3^ (Letunic and Bork, 2016) was used to display various gene contents with the phylogenetic trees.

### Calculation of the Overall Genome Sequence Relatedness Among the Strains of *B. cereus* and *B. thuringiensis*

Pairwise relatedness values based on whole-genome sequences were calculated using OrthoANIu software for ANI (Yoon et al., 2017b) and GGDC v. 2.1 for dDDH (Meier-Kolthoff et al., 2013).

### Detection of Toxin-Related Genes in *B. cereus* and *B. thuringiensis* Genomes

Bt toxin genes used as references for the genomic analyses were downloaded from the Bt toxin nomenclature database^4^ (Crickmore et al., 1998); the structural classifications of the Bt toxins were taken from an earlier study (Van Der Hoeven, 2014). Other reference pathogenic genes detected in the *B. cereus* group were used as described previously (Kovac et al., 2016). The gene sequences were downloaded from the UniProt database (Apweiler et al., 2004) and additional information drawn from SwissProt (Boutet et al., 2016). The presence or absence of genes homologous to the reference toxins was determined by the tblastn search from the standalone BLAST package (Camacho et al., 2009). Parameters were set as default options except for the *e*-value cutoff 1*e*−5. Hits with ≥70% sequence identity and ≥70% alignment length were used as a cutoff to recognize homologs (Yoon et al., 2017a). In addition, a 78% sequence identity cutoff was applied to pesticidal genes for annotating secondary subgroups (Crickmore et al., 1998).

## Results

### Genomic Characteristics and Relatedness of the *B. cereus* and *B. thuringiensis* Strains

**FIGURE 1:**
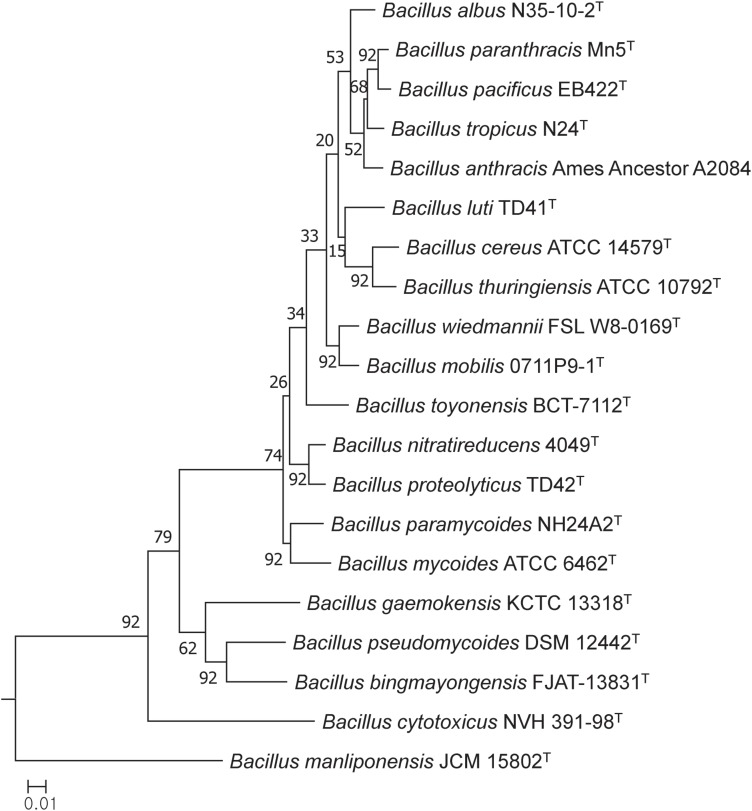
Maximum-likelihood tree based on 92 single copy core gene sequences showing relationships between the type trains of *B. cereus* and *B. thuringiensis* and between them and corresponding strains of other *B. cereus sensu lato* species. The numbers at the nodes are bootstrap values based on 1000 replicates. The bar stands for the number of substitutions per nucleotide.

**FIGURE 2:**
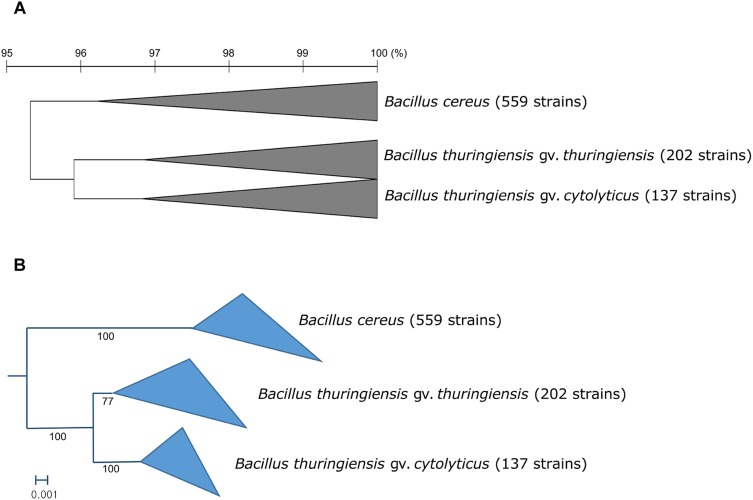
**(A)** Collapsed OrthoANIu dendrogram and **(B)** collapsed phylogenomic tree based on 92 universal bacterial genes showing relationships between the three subclades of the *B. cereus*–*B. thuringiensis* clade. *Bacillus toyonensis* BCT-7112^T^ was used as the outgroup (not shown). The scale bar on the OrthoANIu dendrogram stands for OrthoANIu values (%). Bar on the phylogenomic tree indicates the number of substitutions per nucleotide.

In general, most of the genomes identified as either *B. cereus* or *B. thuringiensis* were found to have genome sizes within the range 5.0–7.9 Mbp while the corresponding G + C content ranged from 33.8 to 35.4 mol% (Supplementary Table S1). It is evident from the phylogenomic tree that the *B. cereus* and *B. thuringiensis* strains are closely related and separated from the type strains of other species belonging to *B. cereus sensu lato* (Figure 1). The ANI and dDDH values between type strains of *B. cereus* and *B. thuringiensis* were 96.71 and 71.20%, respectively (Table 2), that is greater than the species boundary cutoffs (95–96 and 70%, respectively). Within the clade encompassing the *B. cereus* and *B. thuringiensis* strains, three subclades were recognized at the 96% ANI cutoff; these were considered to correspond to *B. cereus*, *B. thuringiensis* genomovar *cytolyticus*, and *B. thuringiensis* genomovar *thuringiensis* (Figure 2A).

**TABLE 2:** Pairwise ANI (Yoon et al., 2017b) and dDDH (Meier-Kolthoff et al., 2013) values between the type/representative strains of three subclades of the *B. cereus*–*B. thuringiensis* clade.

	**1**	**2**	**3**
1		96.71	95.83
2	71.20		96.25
3	66.00	69.10	

*ANI values are on the top-right and dDDH values are on the bottom-left. Strains: 1, *B. cereus* ATCC 14579^*T*^; 2, *B. thuringiensis* gv. *thuringiensis* ATCC 10792^*T*^; and 3, *B. thuringiensis* gv. *cytolyticus* NCTC 6474.*

### Phylogenomic Overview of *B. cereus* and *B. thuringiensis*

The topology of the genome-based phylogenetic tree based on universal bacterial core genes showed that the analyzed strains could be assigned to three subclades (Figure 2B). This result indicates that the current taxonomic annotation in public databases is incorrect because strains annotated as either *B. cereus* or *B. thuringiensis* are irregularly positioned across the phylogenetic tree.

### The Absence of Five Genes Involved in Emesis

It can be seen from the genomic mining data that five genes (*cesA*, *cesB*, *cesC*, *cesD*, and *cesE*) involved in producing the vomiting-induced endotoxin cereulide (Ezzell and Welkos, 1999) were absent from all of the analyzed genomes. In contrast, two transcriptional regulators involved in activating emesis, namely *abrB* and *codY* (Lucking et al., 2015), were universally present (Supplementary Table S2).

### Distribution of *B. cereus* Toxin Genes

**FIGURE 3:**
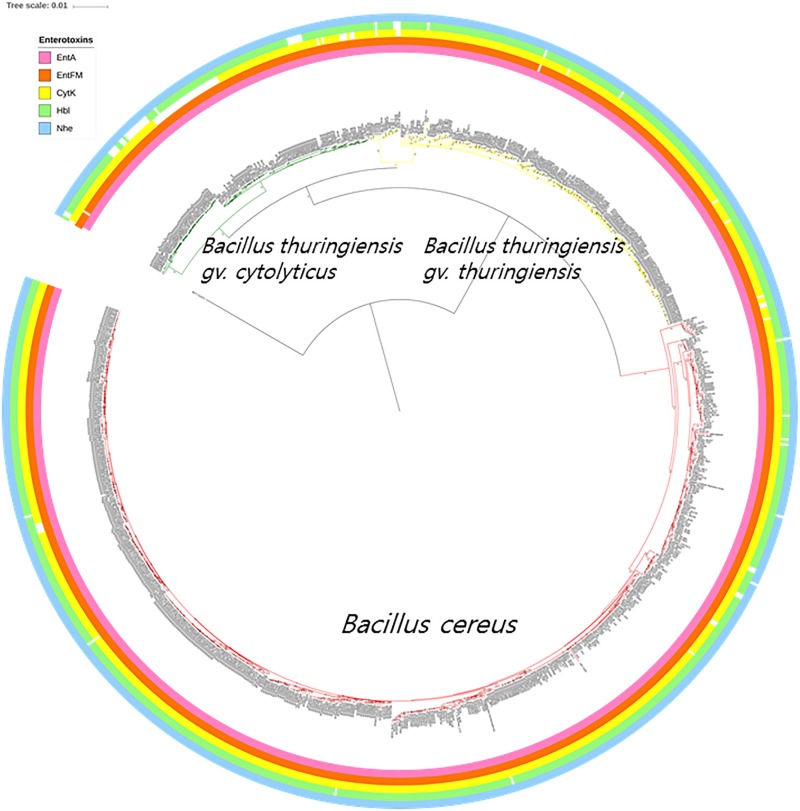
The bacterial core gene-based phylogenomic tree of *Bacillus cereus* and *Bacillus thuringiensis* strains together with labeled enterotoxin genes. The numbers at each branching point are bootstrap values. Bar indicates the nucleotide substitution rate per site. *Bacillus toyonensis* BCT-7112^T^ was used as the outgroup. Taxonomic groups are indicated in different colors: red, *B. cereus*; yellow, *B. thuringiensis* gv. *thuringiensis*; and green, *B. thuringiensis* gv. *cytolyticus*. Genes are displayed on the outer circles: EntA, enterotoxin A; EntFM, enterotoxin FM; CytK, cytotoxin K; Hbl, hemolysin BL; Nhe, non-hemolytic enterotoxin.

Multiple types of diarrheal causative toxins are formed by members of the *B. cereus* group, such as those expressed by operons (*nhe* and *hbl*) and genes (*cytK*, *entA*, and *entFM*); those present in the *nhe* operon were found in all but three of the genomes, namely strains GeD10, BGSC 4BW1, and SJ-S28. Similarly, four genes (*hblA*, *hblB*, *hblC*, and *hblD*) belonging to the *hbl* operon were completely absent from 29 out of the 898 strains belonging to the *B. cereus*–*B. thuringiensis* clade. The genomes from most of the strains carried a single gene associated with enterotoxin production; 898 *entA* genes, 843 *cytK* genes, and 896 *entFM* genes. In addition, only four genomes lacked *cerA*, one *cerB*, and four *clo* (Supplementary Table S2). The distributions of *B. cereus* toxin genes among the strains of *B. cereus–B. thuringiensis* clade were visualized in the phylogenomic tree (Figure 3).

### The Absence of Anthrax-Related Genes

Genes associated with the production of anthrax toxin (*atxA*, *cya*, *lef*, and *pagA*) were not detected in the genomes of the *B. cereus* and *B. thuringiensis* strains even though some of them possessed two capsule forming genes (Supplementary Table S2). Also, the analyzed strains do not have the capsular synthesis regulator genes *acpA* and *acpB* (Drysdale et al., 2004). This suggests that the capsule formation genes may not be expressed unless an unknown transcription factor reacts with them (Supplementary Table S2).

### The Intermittent Distribution of Bt Toxins in the Phylogenomic Tree

**FIGURE 4:**
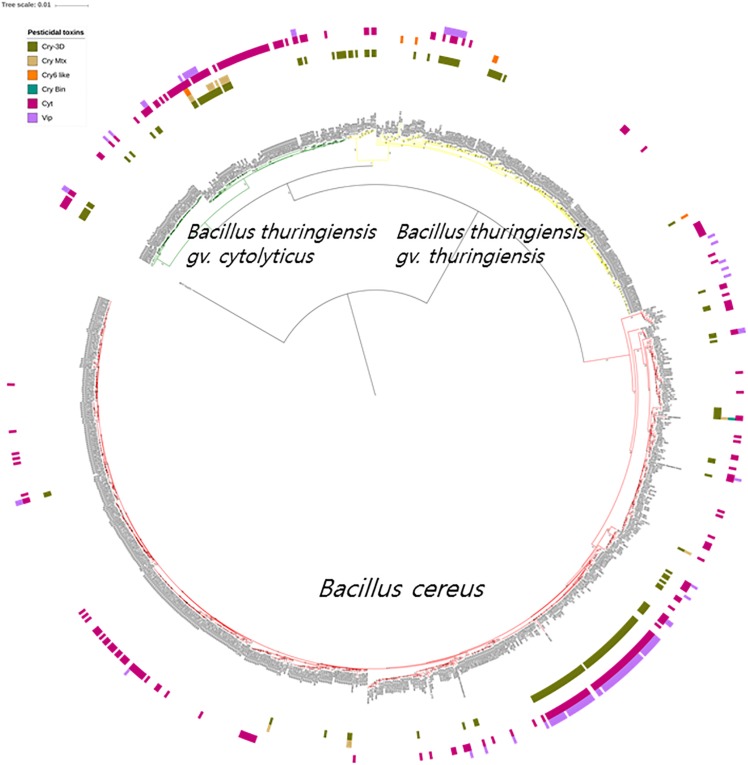
The bacterial core gene-based phylogenomic tree of *Bacillus cereus* and *Bacillus thuringiensis* strains together with labeled pesticidal genes. The numbers at each branching points are bootstrap values. Bar indicates the nucleotide substitution rate per site. *Bacillus toyonensis* BCT-7112^T^ was used as the outgroup. Taxonomic groups are indicated in different colors: red, *B. cereus*; yellow, *B. thuringiensis* gv. *thuringiensis*; and green, *B. thuringiensis* gv. *cytolyticus*. Genes are displayed on the outer circles: Cry 3D, insecticidal crystal protein with three protein domains; Cry Mtx, insecticidal crystal protein that sequentially resembles the toxin of *Clostridium perfringens*; Cry Bin, a conjugated insecticidal crystal protein that sequentially resembles the toxin of *Lysinibacillus sphaericus*; Cry6 like, Cry toxins with shorter sequence and distinct structures; Cyt, insecticidal cytotoxic protein; Vip, insecticidal vegetative protein.

It is apparent from the genomic data that Bt toxin genes are present in strains distributed across the three subclades though unlike the diarrheal toxin operons (*nhe* and *hbl*) they do not predominant in these subclades (Figure 4). Cry genes with three domains (Cry-3D) were prevalent compared with other types of Cry genes. It can also be concluded from the phylogenetic analysis that Cry-3D genes have frequently been transferred among members of the *B. cereus* group, especially toxins belonging to the Cry1 and Cry2 groups (Supplementary Table S2). Some of the Cry toxins detected in the genomes were found to have distinct structures. Cry toxins homologous to the Mtx toxin of *Clostridium perfringens* were detected in 15 strains while seven strains were shown to contain a distinct structure and shorter sequences of the Cry toxin (Cry6, Cry22, and Cry37; named as “Cry6-like toxins”). Only strain 62 contained a Cry toxin homologous with the toxin of *Lysinibacillus sphaericus* (Cry-Bin). Further, some strains contained a pair of Cry proteins that acted as conjugative toxins. Two neighboring strains were found to have the Cry23–Cry37 pair (BGSC 4AA1 and BGSC 4BR1) and strain 62 possesses a Cry34–Cry35 pair. It is particularly interesting that Cyt, Vip, and other types of pesticidal toxins were associated with the genomes of strains spread across the phylogenetic tree (Figure 4).

## Discussion

This study was undertaken to try and resolve the confusion over the taxonomic status of *B. cereus* (Frankland and Frankland, 1887) and *B. thuringiensis* (Berliner, 1915), taxa of importance to agriculture, industry, and medicine (Bottone, 2010; Bravo et al., 2013; Lacey et al., 2015; Hong et al., 2016; Kumari and Sarkar, 2016; Soni et al., 2016; Raymond and Federici, 2017). The outcome of the study together with those of previous phylogenomic analyses of species classified as *B. cereus sensu lato* (Liu et al., 2015, 2017b, 2018) is a timely reminder that advances in the classification, nomenclature, and identification of prokaryotes reflect the introduction of new metrics for the recognition of species and genera (Chun and Rainey, 2014; Chun et al., 2018) which are based on better quality taxonomic data drawn from the application of new technologies (Goodfellow et al., 2014). This study also provides further evidence that analyses of high-quality genome sequences provide a framework for the clarification of taxonomically complex groups of prokaryotes (Carro et al., 2018; Sangal et al., 2018). Further, genome-based taxonomic investigations have proved clarifying the status of taxa of biotechnological, ecological, and medical importance, as exemplified by work on the phylum Actinobacteria (Nouioui et al., 2018) and more specifically the genera *Amycolatopsis* (Sangal et al., 2018) and *Mycobacterium* (Gupta et al., 2018).

The phylogenetic tree based on core genome sequences shows that the type strains of *B. cereus* and *B. thuringiensis* form a well-supported clade that can be distinguished from corresponding lineages composed of strains of other validly published species classified as *B. cereus sensu lato*. In general, better resolution was found between the type strains in the core gene tree than in the corresponding 16S rRNA gene tree generated by Liu et al. (2017a).

The genomic screening data underpin and extend those from many previous studies which found that diarrheal and Bt toxin genes cannot be used to distinguish between *B. cereus* and *B. thuringiensis* (Carlson et al., 1996; Helgason et al., 2000; Priest et al., 2004; Rasko et al., 2007; Stenfors Arnesen et al., 2008; Liu et al., 2015; Miller et al., 2018). In the present study, multiple diarrheal toxin genes (e.g., *cytK*, *entA*, *entFM*, *hbl*, and *nhe*) were found in nearly all members of the *B. cereus*–*B. thuringiensis* clade whereas strains with Bt toxin genes were shown to be polyphyletic in the phylogenomic tree (Figures 3, 4). Emetic toxin genes involved in the production of cereulide (Ezzell and Welkos, 1999) were not present in any of the *B. cereus* and *B. thuringiensis* strains thereby providing further evidence that diarrheal and emetic *B. cereus* belong to different evolutionary lineages (Ehling-Schulz et al., 2005; Carlin et al., 2006). In this respect, it is interesting that the reference strain of emetic *B. cereus*, namely AH 187 (=F4810/72) (Rasko et al., 2007) has been shown to be a member of *Bacillus paranthracis*, a species assigned to *B. cereus sensu lato* by Liu et al. (2017a)^5^. In turn, none of the *B. cereus* and *B. thuringiensis* strains contained genes (*atxA*, *cya*, *lef*, and *pagA*) related to those implicated in the production of anthrax toxins (Kovac et al., 2016).

Within the monophyletic clade encompassing the *B. cereus* and *B. thuringiensis* strains, three subclades were consistently recovered by ANI-based hierarchical clustering (Figure 2A) and bacterial core gene-based phylogenetic analysis (Figure 2B), respectively. The subclade that includes *B. cereus* type strain represents the *B. cereus* whereas the two other subclades were designated as two genomovars, namely *thuringiensis* and *cytolyticus* of *B. thuringiensis*. We propose strain NCTC 6474 (NCBI genome accession GCA_900445335.1) as the representative strain of *B. thuringiensis* gv. *cytolyticus*. The two genomovars proposed in this study may be equated with the rank of subspecies and formally described as such when sufficient supporting phenotypic data are acquired. It is noteworthy that genomovars represent genomically coherent taxa at the intra-species level that is not covered by the International Code of Nomenclature of Prokaryotes (Parker et al., 2019), as spelled out by Rule 14a and in Appendix 10.

One of the advantages of genome-based classification is the use of objective criteria in the definition of species. In this study, the ANI and dDDH values found between the type strains of *B. cereus* and *B. thuringiensis* were slightly higher than the generally accepted species boundary cutoffs. Despite this, we decided against combining the two species into single species as the name *B. thuringiensis* has been extensively used in various microbiological disciplines, especially in agriculture and biotechnology. Instead, we have provided a genome-based taxonomic framework where *B. cereus* and *B. thuringiensis* isolates can be identified not by the unreliable biomarkers (e.g., toxin genes) but by objective molecular methods.

## Conclusion

In conclusion, on the basis of large-scale genomic analyses, we propose that *B. thuringiensis* be divided into two genomovars and the isolates of *B. cereus* and *B. thuringiensis* be identified by genome-based methods, but not by phenotypic or genotypic characterization involving insecticidal genes.

### Emended Description of *Bacillus cereus* Frankland and Frankland, 1887

Data are taken from the present study and from the description of *B. cereus* as given by De Vos and Logan (2011).

Gram-stain-positive, facultatively anaerobic, usually motile rods (1.0–1.2 × 3.0–5.0 μm) that occur singly and in pairs and chains; and form ellipsoidal, sometimes cylindrical, subterminal, sometimes paraientral spores in unswollen sporangia; spores may lie obliquely in the sporangia. The sporangia of some strains carry parasporal bodies adjacent to spores. These crystalline protein inclusions, which vary in shape, are found outside the exosporium and are readily separated from liberated spores. The crystalline proteins and cytolytic proteins are prototoxins which may be toxic for certain insects and other invertebrates, including flatworms, mites, nematodes, and protozoa. The ability to synthesize parasporal bodies is plasmid-borne and may be lost on subculture. Cells grown on glucose agar produce large amounts of storage material giving them a vacuolated to foaming appearance. Cells are characteristically large (2–7 μm in diameter) and vary in shape from circular to irregular with entire to undulate, crenate, or fimbriate edges; they usually have matt or granular textures, but smooth and moist colonies may occur. The minimum temperature for growth is usually 10−20°C, and the maximum 40−50°C. Catalase positive and oxidase negative. Voges–Proskauer positive. Citrate is used as a sole carbon source. Endospores are widespread in soil and many other environments. The diarrheal enterotoxins are widely present, but emetic enterotoxins are absent. Genome sizes range from 5.1 to 7.9 Mbp and corresponding DNA G + C values are within the range 33.8–35.4%, based on 559 genome sequences.

Type strain is ATCC 14579^T^ (=DSM 31^T^=JCM 2152^T^=LMG 6923^T^=NCIMB 9373^T^=NRRL B-3711^T^=IAM 12605^T^).

### Emended Description of *Bacillus thuringiensis* Berliner, 1915

Data are taken from the present study and from the description of *B. thuringiensis* as given by De Vos and Logan (2011).

Gram-stain-positive, facultatively anaerobic, usually motile rods (1.0–1.2 × 3.0–5.0 μm) that occur singly and in pairs and chains; and form ellipsoidal, sometimes cylindrical, subterminal, sometimes paraientral spores in unswollen sporangia; spores may lie obliquely in the sporangia. The sporangia of some strains carry parasporal bodies adjacent to the spores. These crystalline protein inclusions, which vary in shape, are found outside the exosporium and are readily separated from liberated spores. The crystalline proteins and cytolytic proteins are prototoxins which may be toxic for certain insects and other invertebrates, including flatworms, mites, nematodes, and protozoa. The ability to synthesize parasporal bodies is plasmid-borne and may be lost on subculture. Cells grown on glucose agar produce large amounts of storage material giving them a vacuolated to foaming appearance. Cells are characteristically large (2–7 μm in diameter) and vary in shape from circular to irregular with entire to undulate, crenate, or fimbriate edges; they usually have matt or granular textures, but smooth and moist colonies may occur. The minimum temperature for growth is usually 10–15°C, and the maximum 40–50°C. Catalase positive and oxidase negative. Voges–Proskauer positive. Citrate is used as a sole carbon source. Endospores are widespread in soil and many other environments. The diarrheal enterotoxin is present in most strains. Strains can be assigned to two genomovars based on genomic relatedness; *B. thuringiensis* genomovar *cytolyticus* and *B. thuringiensis* genomovar *thuringiensis*. The majority of strains belonging to genomovar *cytolyticus* produce cytolytic toxins whereas few of those in genomovar *thuringiensis* do so. Genome sizes range from 5.0 to 7.1 Mbp and corresponding DNA G + C values are within the range 33.8–35.4%, based on 339 genome strains.

Type strain is ATCC 10792^T^ (=CCUG 7429^T^=CIP 53.137^T^=DSM 2046^T^=HAMBI 478^T^=JCM 20386^T^=LMG 7138^T^=NBRC 101235^T^=NCAIM B.01292^T^=NCCB 70008^T^=NRRL HD-735^T^=VKM B-1544^T^).

## Data Availability

Publicly available datasets were analyzed in this study. This data can be found here: https://www.ezbiocloud.net/.

## Author Contributions

IB, MG, and JC derived the idea for the whole project. IB performed the data collection and phylogenomic analysis, and IB, KL, MG, and JC wrote the manuscript. All authors approved the manuscript.

## Conflict of Interest Statement

The authors declare that the research was conducted in the absence of any commercial or financial relationships that could be construed as a potential conflict of interest.

## Footnotes

1 http://www.btnomenclature.info

2 https://www.ezbiocloud.net/tools/ubcg

3 https://itol.embl.de/

4 http://www.btnomenclature.info/

5 https://www.ezbiocloud.net/genome/explore?puid=696
